# 

**Published:** 1840-01

**Authors:** 


### LOUISVILLE MEDICAL INSTITUTE.

### TO CORRESPONDENTS.

We hope our old correspondents of the W. J. of the M. and P. S. and of the L. J. of M. and S. will not forget us in our new enterprise.

Our next number will contain reports of cases treated in the Louisville Hospital, and papers from Dr. Gross and Dr. Monette, the latter on the autumnal fevers of the State of Mississippi.

THE

### WESTERN JOURNAL

OF

### MEDICINE AND SURGERY:

EDITED BY

DANIEL DRAKE, M. D.

PROFESSOR OF CLINICAL MEDICINE AND PATHOLOGICAL ANATOMY,

AND

LUNSFORD P. YANDELL, M. D.

PROFESSOR OF CHEMISTRY AND PHARMACY,

IN THE

LOUISVILLE MEDICAL INSTITUTE,

WILL be published monthly by the undersigned. Each number will consist of eighty pages, making two large volumes annually. Much of the best professional talent in the Western country is pledged to the support of the work; and with the extensive arrangements made for embracing in each number a summary of the most recent medical intelligence, foreign as well as domestic, the subscribers feel confident, that they will be able to render it a valuable repository of mediical science. One such work, at least, the West certainly owes it to itself to sustain, and the Western Journal of Medicine and Surgery, the Publishers hope, will be found worthy of the liberal patronage of the Medical profession.

TERMS—*Five Dollars*, per annum, payable on the receipt of the second number.

PRENTICE & WEISSINGER,

*Publishers and Proprietors.*

